# Monitoring Vehicle Pollution and Fuel Consumption Based on AI Camera System and Gas Emission Estimator Model

**DOI:** 10.3390/s23010312

**Published:** 2022-12-28

**Authors:** Manuel Rodriguez Valido, Oscar Gomez-Cardenes, Eduardo Magdaleno

**Affiliations:** Department of Industrial Engineering, University of La Laguna, 38200 San Cristóbal de La Laguna, Spain

**Keywords:** sustainability, AI, emission model estimation, MOVESTAR, speed estimation, homography, YOLO

## Abstract

Road traffic is responsible for the majority of air pollutant emissions in the cities, often presenting high concentrations that exceed the limits set by the EU. This poses a serious threat to human health. In this sense, modelling methods have been developed to estimate emission factors in the transport sector. Countries consider emission inventories to be important for assessing emission levels in order to identify air quality and to further contribute in this field to reduce hazardous emissions that affect human health and the environment. The main goal of this work is to design and implement an artificial intelligence-based (AI) system to estimate pollution and consumption of real-world traffic roads. The system is a pipeline structure that is comprised of three fundamental blocks: classification and localisation, screen coordinates to world coordinates transform and emission estimation. The authors propose a novel system that combines existing technologies, such as convolutional neural networks and emission models, to enable a camera to be an emission detector. Compared with other real-world emission measurement methods (LIDAR, speed and acceleration sensors, weather sensors and cameras), our system integrates all measurements into a single sensor: the camera combined with a processing unit. The system was tested on a ground truth dataset. The speed estimation obtained from our AI algorithm is compared with real data measurements resulting in a 5.59% average error. Then these estimations are fed to a model to understand how the errors propagate. This yielded an average error of 12.67% for emitted particle matter, 19.57% for emitted gases and 5.48% for consumed fuel and energy.

## 1. Introduction

Humanity has had the objective throughout the history of reaching the highest levels of prosperity, which has led it to develop great transformations. These transformations have been vertiginous since their beginning in the industrial revolution [1] and have led to a series of positive consequences, such as the increase in wealth and the standard of living of citizens. On the other hand, advances in industry have caused worrying environmental degradation, the concentration of greenhouse gases that affect climate change, noise in cities, overpopulation, unsustainable consumption habits and other pressures on the limited capacity of the earth’s resources [2,3].

Pollution affects not only the planet. Noise and gases have a direct influence on people’s health. Noise pollution in cities has adverse effects ranging from difficulty sleeping, resting, speaking, reading or concentrating to severe physiological and psychological damage [4,5,6,7,8,9]. On the other hand, gases such as ozone (O_3_), sulphur dioxide (SO_2_), nitrogen oxides (NO_x_), carbon monoxide (CO), as well as particulate matter (PM) cause diseases such as lung cancer, pneumonia, asthma, chronic bronchitis and chronic lung diseases [10,11].

With regard to air pollution, it has become a priority problem for decades and an issue to be resolved for governments around the world. Consequently, historical initiatives have been produced, such as the Geneva Convention of 1979 [12], the Kyoto Protocol in 1997 [13] and Paris in 2015 [14], among others. All these initiatives seek to promote policies at the international level to reduce emissions of harmful gases into the atmosphere. All these policies are trying to be carried out in order to achieve what has been called environmental sustainability [15]. Currently, the UN has a 2030 Agenda for sustainability [16]. 

Transport is the largest source of greenhouse gas (GHG) emissions in the EU (27%) and the only sector where current emissions are higher than in 1990 [17]. While cars by themselves are the largest contributor (43.9% of transport emissions), heavy transport has a higher combined impact at 46.2%.

In order to reach the 1.5 °C global temperature rise limit, net anthropogenic greenhouse gas (GHG) emissions must approach zero by 2050 [18]. Currently, decarbonisation pathways in different sectors, such as power generation, light transport and domestic heating/cooling, are relatively well established. However, emissions from heavy industry, agriculture and heavy transport (trucks, buses, shipping and aviation) are more difficult to eliminate [19]. However, the decarbonisation of the transport sector as a whole will influence efforts to reduce emissions to the 2050 limits.

Developing new ways to control and monitor air pollution, improve people’s health and strictly comply with current and future vehicle emission standards represent a major challenge for humanity and for researchers to make the world more sustainable. In this sense, it is very important to closely monitor the sources of emissions, in particular from vehicles. Such monitoring can facilitate the adoption of effective control measures where necessary, as well as the prediction of the impact of transport emissions on ambient air quality [20].

Methodologies for monitoring vehicle emissions onboard and in the laboratory under real-world conditions have contributed to improved tools and computational models for estimating vehicle emissions. Many databases generated using this methodology have been used as input in modelling the traffic-related air quality impact [21]. Although the use of these techniques is one of the most accurate methods for estimating emissions, it is still a technique that is performed under laboratory conditions, i.e., it is not capable of capturing the real-world conditions where the vehicle operates, such as vehicle type, age, emission control devices and operating conditions; urban road type and condition; vehicle maintenance frequency; fuel type; ambient conditions (temperature and humidity); and traffic conditions [22]. Even though standard driving cycles are followed in dynamometer test methods attempting to replicate real-world driving conditions closely, they may not necessarily represent actual real-world driving conditions and emissions thereof. Therefore, in recent years, significant scientific efforts have been directed to measure and analyse real-world driving emissions from vehicles to measure unveiling trends of actual transport emissions.

Under real-world conditions, the measurement methodologies are complex in nature and non-repetitive; however, the emissions inventories developed by the real-world measurements are quite valuable because they can meaningfully contribute to developing emission computation models, to their validation, as well as to identifying gaps between modelling results and real-world driving emissions [20]. The estimation and prediction of vehicle emissions using model-based methods are useful for planning the fleet according to emission standards, developing an emission inventory, monitoring and modelling urban air quality, and assessing the impact of emissions on the environment and human health. 

Model-based emission estimation methods fall into two categories, the macroscopic modelling approach and the microscopic modelling approach [23]. The most important macroscopic models are the traditional Mobile Source Emissions Factor (MOBILE6.2) from the US federation [24], the EMission FACtors (EMFAC2021) from California [25] and the European COPERT-IV [26,27]. These models tend to generate results more quickly but have accuracy problems, as they ignore microscopic vehicle movements, resulting in incorrect estimation of emission rates.

Microscopic models, however, take into account the movement of individual vehicles in space and time, i.e., they use the speed or acceleration data of each vehicle and can therefore generate data correlated with driving behaviour. Among the various microscopic emission models, which are preferentially used to predict emission rates in urban areas, we can find the Comprehensive Modal Emission Model (CMEM) [28], the Virginia Tech Microscopic Emission Model (VT-Micro) [29], the VERSIT+ model [30] and the MOVES model [31]. From the point of view of performing a reliable simulation of driving dynamics, there is a preference for the use of MOVES and ARTEMIS models over others for estimating vehicle emissions in urban areas [31].

With regard to Spain, the current regulations are intended to establish the bases for the prevention and reduction of air pollution. It is aimed at the emission of fourteen pollutants, among which are those mentioned above, and encompasses both concepts related to air quality and aspects of light and noise pollution [32,33]. Within this field, research projects are promoted that seek to reduce emissions of pollutant gases into the atmosphere. Specifically, the research project in which this work is framed aims to contribute to the improvement of the sustainability of the Canary Islands Sea Ports. One of the tasks of this research project is to estimate the gas emissions produced by road traffic in the interior of the ports. With the data obtained, it will be possible to carry out a feasibility study of the electrification of port vehicles, both maritime and terrestrial.

In this work, and as the main objective, we propose a system for estimating vehicle emissions and fuel consumption. The system is based on a camera sensor placed on strategic roads and an artificial intelligence (AI) algorithm to feed a model-based estimator of fuel consumption and gas emissions. The outputs of this AI system, speed and vehicle category, are used as inputs to the model-based emission and consumption approach.

In order to test the AI monitoring proposed method, we used MOVESTAR [34] as a consumption and emission model-based approach. This model is a simplified version of MOVES described in [34]. Specifically, MOVESTAR is an open-source model to calculate the fuel consumption and pollutant emissions of motor vehicles; it is very useful due to its interoperability with other software that allows the authors of this work to implement a pipeline integrating AI and perform benchmarks. The MOVESTAR model only takes the second-by-second vehicle speed data and vehicle type as inputs, without the consideration of geographical location, road grade and other parameters which were included in MOVES. MOVESTAR model approach has been integrated into a traffic simulator such as PTV Vissim [35].

MOVESTAR method needs, as input, an estimation that is as reliable as possible of the speed of the vehicles second-by-second and vehicle category (light or heavy vehicle). The model gives as output results the second-by-second values in grams of CO, HC, NO_x_, CO_2_, fuel, PM_2.5_ elementary and organic carbon emissions, as well as the energy in KJ and fuel consumption [34].

The rest of the present work is organised as follows: Section 2 explains the proposed system implementation. Section 3 details the most relevant results. Finally, conclusions and future work are presented in Section 4.

## 2. System Implementation

A completely passive system is proposed in this work. This approach was chosen because it allows for taking advantage of hardware that, in many cases, is already installed or requires a cheap and non-invasive installation. By combining already in-place hardware with modern software, a new pollution sensor can be achieved.

The goal of this system is to be able to measure the pollution and fuel consumption of cars traversing the road, maintaining a low cost while having an error that is as close as possible to those of active systems such as the ones based on LIDAR. The camera sensor observes a scene with vehicles moving through a road and outputs an estimation of the air pollution produced by each vehicle.

Places such as ports, train stations, medical centres and roads already have a camera surveillance system whose image feed can be reused for our purposes. Thus, giving the parties that own the surveillance system new data that can be used to obtain insight into the amount of pollution in the area. Systems such as the ones described in these works can be complemented by the proposed sensor [36,37].

The sensor consists of hardware and a software module. Two possible versions of the sensor can be implemented, depending on the availability of an already implemented video transmission. In the first version, designed for the case of not having a video transmission, the hardware consists of a camera, a processing unit capable of running computer vision IA algorithms and a wireless communication unit. In the second version, wireless video transmission would be taken advantage of, and the algorithms would run on a server. This latter version is easier to implement, but this work focuses on the more general first case.

The design was made to attend to the fact that sending only a slightly compressed video stream is expensive and power-consuming. Compressing the video too much would result in a loss of precision. A standard surveillance video stream with a resolution of 1080p at 30 fps has a bandwidth consumption of ~5 Megabits/s. Sending the pollution data instead is much cheaper since only a 64-bit datum is required by vehicle, resulting in a stream that is 6 orders of magnitude smaller.

Therefore, a decision to process the video in-place was taken. The downside of this is that it created the need to implement highly optimised software that runs on edge computing devices in a real-time fashion.

### 2.1. Overall System Description

The software consists of three parts:

1. Vehicle localisation and classification;
2. Screen to world coordinate transform;
3. Speed estimation.

The camera captures a continuous image sequence that shows the vehicles traversing a particular road. Each frame of the sequence is fed to a convolutional neural network whose task is two-fold: locating the position of each vehicle in the image and classifying the type of vehicle.

The screen coordinates of the locations are converted into world coordinates within the road plane using a homography matrix. This new set of coordinates is in meters, and therefore we can estimate the distance between each pair of coordinates.

Then, an algorithm takes the output of each frame of the neural network and creates a path for each vehicle composed of all the locations of all the frames where the same vehicle was detected. The distance between the points of the path and their time point yields a speed estimation for each vehicle.

**Figure 1:**
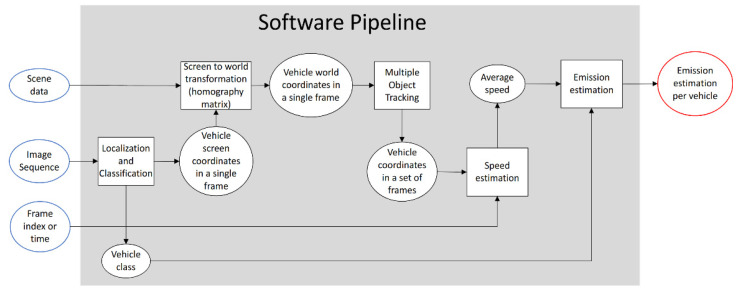
Diagram representing the logical structure of the pipeline that takes a sequence of images and computes an estimate of emissions for each vehicle.

The speed estimation and the class of the vehicle are fed to an emission model that gives an estimate of the gases emitted by the vehicle in the observed trajectory. This pipeline architecture is represented in Figure 1. Square shapes represent processes, while round shapes represent data. Shapes with blue borders represent inputs, and shapes with red borders represent outputs.

Currently, image-based vehicle speed estimation is a booming field of research, and many publications can be found in the specialised literature [38]. Firstly, the vehicles in a scene that is static in most cases must be detected (except images taken by drones or helicopters). The first group of methods consists of subtracting the background of the scene or detecting vehicle license plates [39,40,41,42]. These are the simplest and most classic methods used. However, the current trend seems to point to the use of artificial intelligence, that is, learning-based methods to recognise objects (in this case, vehicles) [43,44,45,46,47,48]. These methods include tracking objects. Once the objects in the scene have been detected, the distance and speed must be estimated, taking into account that an image is a projection on a 2D plane of a three-dimensional image. Currently, the specialised literature calculates distances using three methods: the first is based on the addition of lines or areas that are used to detect the intrusion of the vehicle within the road [49,50,51]. The second method uses homography techniques, usually to transform the scene into a top-down view from which distances can be easily obtained [52,53,54]. A third alternative is used to estimate the distances of objects whose standard size is known, such as car license plates [55,56].

### 2.2. Localisation and Classification

Although convolutional neural networks (CNN) have been used to solve recognition tasks since the 1980s [57], more recent developments have led to great achievements, especially their implementation in GPUs [58]. Because of that, classifying the type of vehicle and localising their position within a single frame is a solved problem for a computing machine. The authors of this work even had the luxury of choosing an adequate network architecture that fits the requirements of this system.

**Figure 2:**
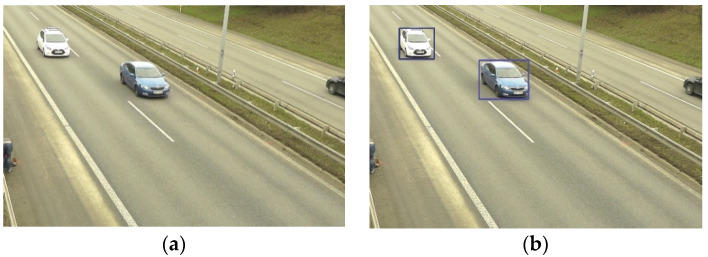
Object localisation performed by YOLOv5 nano: (**a**) input image; (**b**) the result bounding boxes over the vehicles.

For the task of choosing an adequate architecture, the question of what kind of output data are needed must be answered first. The first requirement is that the convolutional neural network should output a vehicle class that is used later to feed the emission model. Examples of these classes are cars, motorbikes and trucks. A neural network that is capable of this is called an “image classifier”. The second requirement is to obtain a set of coordinates that define a bounding box that delimits the area of the image where the classified object was found (see Figure 2). This is called “object localization”. Moreover, the task of finding and detecting the bounding box of objects belonging to specified classes is called “object detection”. There are other kinds of networks that not only find the bounding box where an instance lies but also classify each pixel of the image. This problem does not require this latter type.

After finding the type of CNN that is needed to solve this part of the problem, the authors analysed the best networks in the literature. A good summary of these can be found in [59].

Good candidates that were considered when designing this part of the pipeline were R-CNN, YOLO and RetinaNet. By taking into consideration factors such as speed, quality, ease of use, RAM consumption and portability, the authors focused their analysis on the YOLO family, choosing finally the YOLOv5 architecture [60] and the nano variant, which resulted in sufficient quality and a quite short inference time. The implementation and weights used are made available by the authors. In addition, architecture and weights are available in the torch hub [61].

### 2.3. Screen-to-World Coordinates Transform

Correctly estimating vehicle coordinates impacts the quality of the speed estimation directly, so an accurate solution to this problem is crucial for the achievement of the goals of this work. Other methods estimate the location of the vehicle by using clues such as the size bounding box, the width of the vehicle, the detection of parts such as the plate, etc. To avoid the inherent error that all these methods have, the authors used a coordinate transform based on measured markers in the image.

Two different approaches were explored to produce a solution. The first one consists of using the extrinsic and intrinsic parameters of the camera and the size of the vehicle detection to convert the pixel coordinates into world coordinates. There are two problems with this approach. The camera parameters are not always available. More importantly, the distance of the vehicle to the camera is calculated by comparing the size of the detection to a template vehicle size. This is a very error-prone approximation. 

**Figure 3:**
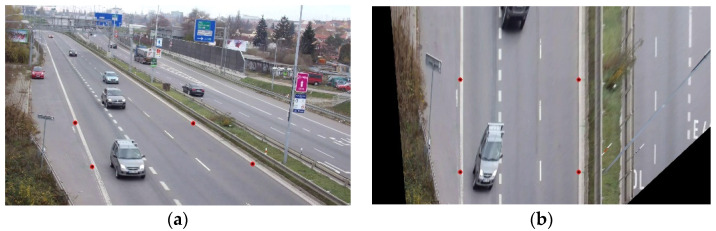
Coordinates transform: (**a**) an input image with the 4 points marked; (**b**) the result of applying the transformation to all the pixels of the image. Note that the road has lost its perspective, and it looks like it is seen from above, while the cars are deformed because they do not belong to the characterised road plane.

The other approach is to manually label four points in the scene once the camera is fixed. By having the relative world coordinates between these points and the absolute screen coordinates in pixels, a homography matrix can be automatically calculated by using the least squares method. In Figure 3, the four points for which their world coordinates and screen coordinates are characterised are used to calculate a homography matrix. This allows for any screen coordinates to be transformed into world coordinates, and therefore their distance can be measured in meters. Additionally, we can obtain screen coordinates from world coordinates by using the inverse matrix.

### 2.4. Multiple Object Tracking

Bounding boxes of the same vehicle along different successive frames must be grouped in a set. For that, an algorithm is needed. This type of problem has been called “Multiple Object Tracking” (MOT) in the literature [62]. YOLO networks are usually paired with a Deep Learning based algorithm, DeepSort [63]. The authors deemed this natural choice to be inadequate for our problem since the RAM consumption was too big, and the inference time ranged between 50 and 150 milliseconds on a small GPU.

The authors could then have used the SORT [64] algorithm, but a more concise approach was used instead. This problem has a set of constraints that can be taken advantage of to better design an ad hoc algorithm instead of a general-purpose MOT algorithm. The vehicles to be analysed have a constrained trajectory; therefore, the set of constraints that were used are as follows:

- The lanes of the road are normally traversed in a single direction, so a vehicle may not travel backward with respect to the direction of the road. Moreover, the angle of travel can differ by a small amount with respect to the angle defined by the direction of the road;
- A vehicle can only change its angle of travel in small increments;
- A vehicle can only travel within a range of speeds;
- A vehicle will finish its trajectory when it crosses the goal line. The goal line is the line perpendicular to the angle of the road that is closer to the end of the road, i.e., the line that intersects the vehicle when it exits the visible part of the road.

With that in mind, the authors designed an algorithm, shown in Algorithm 1, that is capable of taking the set of detections (world coordinates) per frame and instance and sorting them into sets belonging to the same vehicle. Each set is a vehicle trajectory at the end of the. This algorithm, implemented in Python, runs in less than a millisecond per frame in an Intel i5 5500.
**Algorithm 1** Multiple Vehicle Tracking algorithm pseudocode. 1: detections_per_frame contains an array per frame. It is ordered from most recent to least recent. 2: detections_per_frame[i] is an array that contains a 2D world coordinate per vehicle. 3: frame_margin is used to allow the vehicle to disappear from the scene, so all the trajectories can be found by the algorithm. 4: 5: **procedure** MVT 6:   output ← ∅ 7:   **for** frame_idx = frame_margin, **len**(detections_per_frame) **do** 8:     **for** detection_idx = 0, **len**(detections_per_frame[frame idx]) **do** 9:       detection ← detections_per_frame[frame_idx][detection_idx] 10:       **if** detection.y >= (goal_line_y) **then** # This vehicle has crossed the end of the road. 11:         trail_up ← FIND_TRAIL(frame_idx, detection, up) 12:         # Remove each element of trail up from detections per frame 13:         trail_down ← FIND_TRAIL(frame_idx, detection, down) 14:         # Remove each element of trail down from detections per frame 15:         trail ← **concatenate**(trail_up, trail_down) 16:         output.append(trail) 17:       **end if** 18:     **end for** 19:   **end for** 20:   **return** output  21: **end procedure** 22: 23: **procedure** FIND_TRAIL(frame_idx, position, direction) 24:   trail ← ∅ 25:   max_skips← 5 26:   maxdistance ← max_meters_in_frame_vertical 27:   current_frame ← frame_idx 28:   current_position ← position 29:   **while** max_skips > 0 **do** 30:     **if** direction = up **then** 31:       current frame = current frame − 1  32:     **else** 33:       current frame = current frame + 1 34:     **end if** 35:     min_distance_idx ← −1 36:     min distance ← ∞ 37:     **for** detection_idx = 0, **len**(detections_per_frame[current_frame]) **do** 38:       distance ← **compute_distance**(detection, current_position) 39:       distance_x ← detection.x – current_position.x 40:       **if** (distance < min_distance) **and** 41:           ((direction = up) **and** (detection.y > current position.y) **or** 42:            (direction = down) **and** (detection.y < current position.y) **and** 43:           (distance_x < max_meters_in_frame_horizontal) **then** 44:         min_distance_idx ← detection_idx 45:         min_distance ← distance 46:       **end if** 47:     **end for** 48:     **if** min_distance_idx = −1 **and** min_distance < max_distance **then** 49:       max_skips ← 5 50:       max_distance ← max_meters_in_frame_vertical 51:       current_position ← detections_per_frame[current_frame][min_distance idx] 52:       trail.**append**([current_frame, min_distance_idx]) 53:     **else** 54:       max_skips ← max_skips − 1 55:       max_distance ← max_distance ∗ 2 56:     **end if** 57:   **end while** 58: **end procedure**

**Figure 4:**
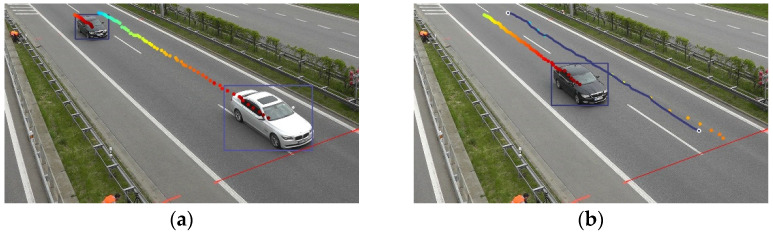
The age of the detection has been colour coded using the jet colormap: (**a**) A car is about to cross the goal line; (**b**) it has already crossed and the MVT algorithm has correctly found the detections that describe its trajectory, allowing it to measure the speed.

When this algorithm is integrated into the system, the flow works as follows. In each frame, every car has been detected and located by a bounding box. The bounding box centroid of each detection constitutes a point that is transformed into world coordinates. When caching these coordinates and their age along the frames of the video, trajectories are formed. The sequence of these world coordinates is fed to the Multiple Vehicle Tracking (MVT) algorithm, which outputs a trajectory for each vehicle. This process is described in Figure 4.

## 3. Results

Three approaches were considered with the goal of assessing the quality of the system implemented. One of them is to create a dataset containing labelled videos with the speed and emissions per vehicle. This approach is very costly both in terms of time and economics and is used in the future for further improvements. The second approach is to create a synthetic dataset. The advantage of this is that the ground truth data are perfectly accurate and trivial to obtain. The downside is that the images would be generated by a renderer which is not always very realistic compared to real images obtained with a camera.

The third approach was to use an existing dataset that was made available to the authors of this work: the BrnoCompSpeed dataset [65]. This dataset consists of 21 full-HD videos (1920 × 1080 @ 100 fps) of one hour each, captured at six separate locations, showing a total of 20,865 vehicles traversing a road. Their speed was measured using LiDAR, and the quality of their measurements was verified by using GPS tracks.

### 3.1. Speed Estimation Error

This data set contains a good amount of information that is enough to assess the quality of our speed estimation. The whole dataset was run through the described system, yielding the following results that are compared with the same algorithms that were discussed in [66]:

- FullACC is a fully automated system where the vanishing points and lanes are inferred by the trajectory of the vehicles, and the scale is inferred by applying a statistical model of the size of the car on the detected bounding box of each vehicle;
- OptScale maintains the vanishing points calculated by FullACC and calculates the scale using the information of the vanishing point following the direction of the car, calculated by min eigenvector detector and KLT tracker;
- OptScale VP2 is the same as OptScale, but the vanishing point has been calculated by measuring the orientation of strong edges perpendicular to the first vanishing point;
- OptCalib keeps the first vanishing point from FullACC and uses the known second vanishing point, and computers scale from lengths in direction to the first vanishing point;
- OptCalib VP2 keeps the first vanishing point from FullACC and uses the known second vanishing point, and computes scale from lengths in direction to the second vanishing point.

Table 1 shows the results yielded by the system proposed in this paper compared to the results obtained in [66].

**Figure 5:**
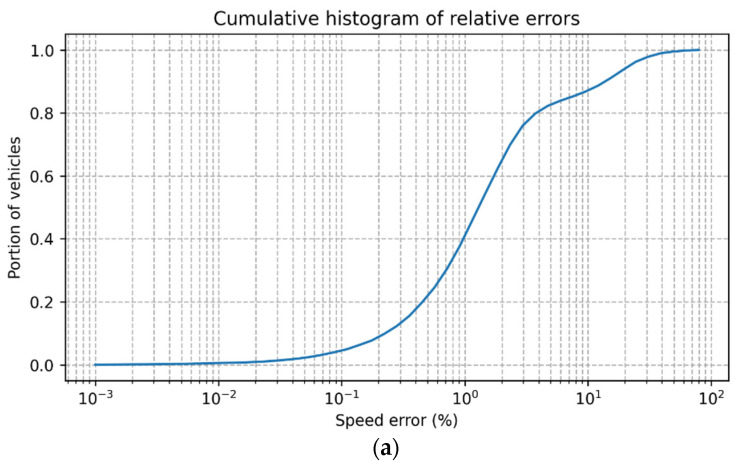

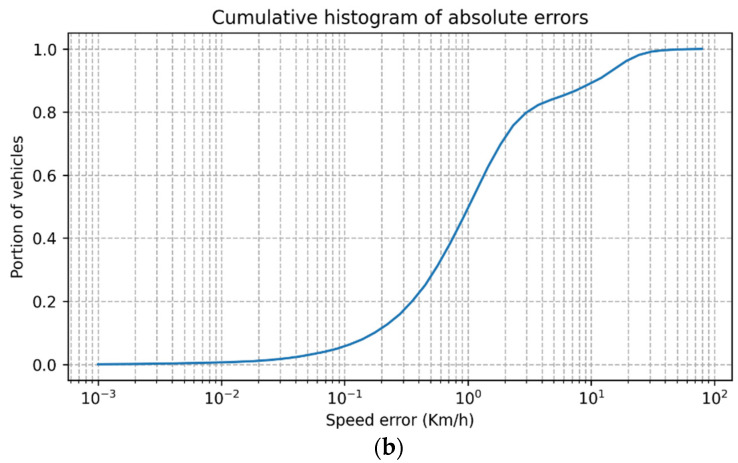
Cumulative histograms that show the distribution of the speed error (logarithmic scale) when compared to the ground truth: (**a**) plot of relative errors; (**b**) plot of absolute errors.

Figure 5 shows the distribution of errors when executing the system on the BrnoCompSpeed dataset. The speed estimation results show that our system is comparable to the partially automated results of [64] and is better than the ones that use the second vanishing point to compute scale. For example, 80% of the vehicles had an absolute speed error (Figure 5b) smaller than 3 km/h, or 3.8% of relative error (see Figure 5a). Moreover, the measured error is small enough to fit the purposes of this work, and more importantly, our system is fit to be run on an edge device with constrained processing power and RAM. This last requirement was not one of the goals of the systems evaluated in [66] but is of utmost importance in this work.

### 3.2. Emission Estimation Error

Once the bounds of the error of the speed estimation are calculated, a propagation of them is performed to obtain the theoretical bounds of the error by using the MOVESTAR model. The results can be seen in Table 2.

**Figure 6:**
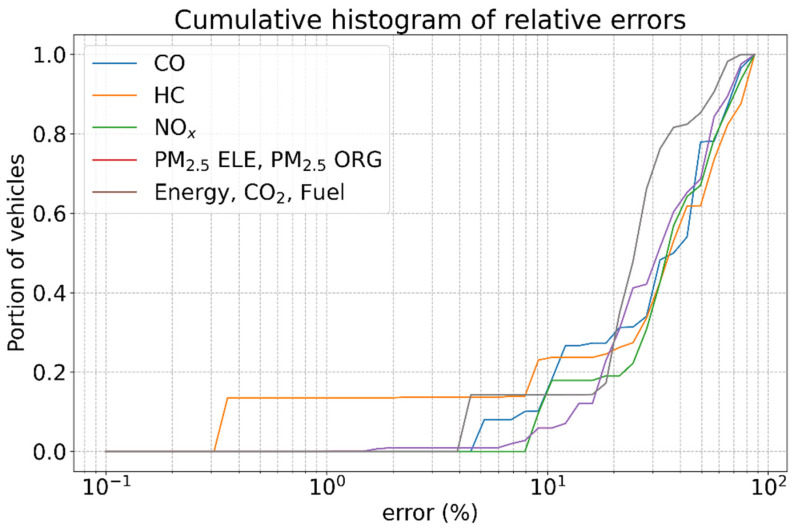
Cumulative histograms of errors of emission estimations based on the speed estimations computed by our system.

The distribution of the error for each estimated variable is depicted in Figure 6. The data indicate that there is a discretisation on the inputs, so small variations in the speed return the exact same estimated emission values, resulting in medians of value 0 and steps in the plot. The plot indicates that there is a discretisation on the inputs, so many values of speed return the same estimation values. For NO_x_ estimation, 50% of total vehicles have an error of less than 35% compared to the estimation of the ground truth speed. For all variables, 50% of the measurements have less than 37% of the total error. Energy, CO_2_ and fuel have the same error distribution. PM_2_._5_ ELE and PM_2_._5_ have the same error distribution as well. Compared with the speed estimation errors, the errors propagate similarly in the case of energy, CO_2_ and fuel as their relative means, and the 95 percentile are comparable. On the other hand, CO and HC seem to have errors that are about five times those of the speed estimation. In reference [65], we added a link to GitHub, where the source code is available.

## 4. Conclusions and Future Work

We implemented an intelligent system based on emission modelling and a camera to estimate gas emissions and fuel consumption to be used as an estimator of pollution caused by vehicle traffic. The system was tested using the MOVESTAR open-source model, and as a data source, we used the BrnoCompSpeed dataset.

The implemented system is innovative as it only uses, compared to other methods, a camera and a computational AI system to estimate pollutant gases and fuel consumption on road traffic; i.e., the use of an “intelligent camera” as the only sensor to estimate emissions and fuel consumption. This would allow us to use the thousands of cameras already installed on the roads of cities and countries to estimate the emissions inventory.

The error speed estimation of the proposed algorithm improves two of the five algorithms studied (FullACC and OptScale VP2), and with respect to the other three algorithms (OptCalib, OptCalib VP2 and OptScale), the error incurred on the same dataset (BrnoCompSpeed dataset) is of the same magnitude. In the case of the emission estimation stage, the authors do not have ground truth data, so the evaluation was performed by propagating the speed estimation errors through the MOVESTAR model. This yielded an average error of 12.67% for emitted particle matter, 19.57% for emitted gases and 5.48% for consumed fuel and energy.

The AI algorithms were rigorously developed so that the implemented system is ready for future installation on roads. In this sense, its installation in the traffic control camera system will be simple once the future algorithm implementation platform has been chosen, which can be a GPU or FPGA device, for example.

Finally, for future improvements, the authors consider that the implemented system can be integrated into more common or popular models, such as MOVES and COPERT, because they allow for more fine-grained inputs such as more vehicle categories, road type, environmental parameters, etc. In addition, as a possible improvement, the authors will study the obtaining of the homography matrix using a length of road markings, the standard for each country, with the aim of automating this process.

## Figures and Tables

**Table 1:** The results yielded by the system proposed in this paper are compared to the results obtained in [66]. All data are in kilometres per hour or percentage.

System	Absolute Mean	Relative Mean	Absolute Median	Relative Median	Absolute 95 Percentile	Relative 95 Percentile
Ours	4.26	5.59	1.28	1.68	21.85	27.79
FullACC	8.59	10.89	8.45	11.41	17.14	19.84
OptScale	1.71	2.13	1.17	1.51	4.69	5.56
OptScale VP2	15.66	19.83	13.09	17.51	47.86	59.25
OptCalib	1.43	1.81	0.83	1.05	3.89	5.07
OptCalib VP2	2.43	30.8	1.40	1.76	6.66	8.00

**Table 2:** Errors of emission estimations based on the speed estimations computed by our system compared with ground truth data.

System	Absolute Mean	Relative Mean	Absolute Median	Relative Median	Absolute 95 Percentile	Relative 95 Percentile
CO	0.002234 g/s	26.07%	0 g	0%	0.0058 g/s	124.96%
HC	0.000019 g/s	27.16%	0 g	0%	0.000164 g/s	74.013%
NO_x_	0.000079 g/s	16.72%	0 g	0%	0.000625 g/s	57.46%
PM_2.5_ Ele	0.000001 g/s	12.67%	0 g	0%	0.000003 g/s	60.425%
PM_2.5_ Org	0.000003 g/s	12.67%	0 g	0%	0.000023 g/s	60.425%
Energy	3.93 KJ/s	5.48%	0 g	0%	24.053 KJ/s	31.83%
CO_2_	0.28 g/s	5.48%	0 g	0%	1.71 g/s	31.83%
Fuel	0.088 g/s	5.48%	0 g	0%	0.54 g/s	31.83%

## Data Availability

Not applicable.
